# Joint User-Slice Pairing and Association Framework Based on H-NOMA in RAN Slicing

**DOI:** 10.3390/s22197343

**Published:** 2022-09-27

**Authors:** Mai A. Riad, Osama El-Ghandour, Ahmed M. Abd El-Haleem

**Affiliations:** 1 Electronics and Communications Engineering Department, Faculty of Engineering, Helwan University, Helwan 11792, Egypt; 2 Electronics and Communications Engineering Department, Faculty of Engineering, New Cairo Academy University, Cairo 11865, Egypt

**Keywords:** 5G, RAN slicing, HetNet, UE-slice association, UE-slice pairing, eMBB, H-NOMA, uRLLC, matching game

## Abstract

Multiservice cellular in Radio Access Network (RAN) Slicing has recently attained huge interest in enhancing isolation and flexibility. However, RAN slicing in heterogeneous networks (HetNet) architecture is not adequately explored. This study proposes a pairing-network slicing (NS) approach for Multiservice RAN that cares about quality of service (QoS), baseband resources, capacities of wireless fronthaul and backhaul links, and isolation. This intriguing approach helps address the increased need for mobile network traffic produced by a range of devices with various QoS requirements, including improved dependability, ultra-reliability low-latency communications (uRLLC), and enhanced broadband Mobile Services (eMBB). Our study displays a unique RAN slicing framework for user equipment (UE) for joint user-association. Multicell non-orthogonal multiple access (NOMA)-based resource allocation across 5G HetNet under successive interference cancelation (SIC) is seen to achieve the best performance. Joint user-slice pairing and association are optimization problems to maximize eMBB UE data rates while fulfilling uRLLC latency and reliability criteria. This is accomplished by guaranteeing the inter- and intra-isolation property of slicing to eliminate interferences between eMBB and uRLLC slices. We presented the UE-slice association (U-S. A) algorithm as a one-to-many matching game to create a stable connection between UE and one of the base stations (BSs). Next, we use the UE-slice pairing (U-S. P) algorithm to find stable uRLLC-eMBB pairs that coexist on the same spectrum. Numerical findings and performance analyses of the submitted association and pairing technique show they can all be RAN slicing criteria. We prove that the proposed algorithm optimizes system throughput while decreasing uRLLC latency by associating and pairing every uRLLC user in mini slots.

## 1. Introduction

The tremendous recent advances in mobile communication, intelligent user devices, and traffic demand have necessitated cell density optimization in extensive networks to provide high data throughput and low latency [1]. Ultra-dense networks have been considered a promising solution for the fifth generation (5G) networks as network densification can boost network coverage and capacity while reducing operational and capital expenditures. In addition, the backhaul network needs to be enhanced to transmit traffic from base stations (BSs) to the core network (CN) and vice versa, and sub-6 GHz wireless backhaul (WBH) facilitates non-line-of-sight (NLOS) transmission, while minimal delay on radio access and backhaul lines has become crucial for delivering various services and applications, such as Voice over Internet Protocol (VoIP) and online gaming, with an acceptable quality of service in future cellular networks, e.g., VoIP and online gaming (QoS) [2]. However, traditional HetNets dedicate radio resources to UE in time or frequency domains, i.e., orthogonal multiple access (OMA), where the availability of the radio resources strictly limits the number of served UEs at a given time instant. Considering the expected explosive number of devices, massive connectivity necessitates more spectrum-efficient access schemes with extended coverage [3].

On the other hand, non-orthogonal multiple access (NOMA) has recently attracted attention by permitting sharing of the same radio resources among a set of UEs. NOMA can support more users than the number of available orthogonal resources [4], leading to higher spectral efficiency and user fairness compared to standard OMA techniques. The principle of NOMA leverages the concept of superposition coding (SPC) at the transmitter to multiplex users in the power domain and successive interference cancellation (SIC) at the receiver [5].

The 5G network services have been classified into three categories by the International Telecommunication Union (ITU): Enhanced Mobile Broadband (eMBB), Ultra-reliable and Low-latency Communications (uRLLC), and Massive Machine Type Communications (mMTC). The eMBB service focuses on high bandwidth requirement services, uRLLC focuses on latency-sensitive services, and mMTC focuses on services with high connection density requirements [6]. However, those services cannot always be achieved through a common network setting. To allow the coexistence of these heterogeneous services with diverse requirements within the same Radio Access Network (RAN) architecture, the concept of network slicing (NS) has been proposed [7], which slices the network into logical and physical sub-networks, usually with customized requirements in terms of latency, energy efficiency, mobility, massive connectivity, and throughput, aiming at guaranteeing minimum performance requirements and isolation [8]. The network slice comprises RAN and core network [9] to support end-to-end service requirements. This can be performed thanks to network softwarization and virtualization (SDN, NFV), which are considered the primary enabler of Resource as a Service (RaaS) beyond 5G (B5G) [10]. As for RAN slicing, additional challenges are faced due to limited capacity, radio channels, and performance isolation, which is still under development [11].

The uRLLC service is intended for event-driven, mission-critical, and industrial settings that may be helped in achieving QoS criteria such as ultra-low latency and ultra-high dependability. In addition, the standard uRLLC sets rigorous latency and reliability standards, with typical latency and reliability requirements of 1 ms/packet and up to 99% successful packet delivery [12]. Since both the eMBB and the URLLC are critical components of communication traffic in 5G, several researchers have addressed the problem of the coexistence between various services [13]. Some of the main challenges in HetNets are the user association process and the user pairing process. The users must be associated to different base stations (BSs) in the HetNets [14].

Consequently, and motivated by the aforementioned benefits of NOMA and RAN slicing, this paper considers H-NOMA in RAN slicing and studies the problem of the coexistence of services with heterogeneous requirements. User association and pairing algorithms are considered the various types of UEs and their different connection demands in a HetNet.

### 1.1. Related Work

Wireless networks emphasize resource slicing to increase spectrum utilization from NS problems in HetNet, by including user association and resource allocation. Furthermore, most literature saw NS’s constraint-isolated resource allocation as an issue. In [15] Earliest Deadline First (EDF) scheduling, originally used in real-time operating systems, is exploited for radio resource allocation in RAN slicing for the first time.

Multiplexing the traffic from URLLC and eMBB users constitutes a significant challenge in 5G, hence it has been tackled several times in the literature very recently. Diverse QoS demands, in addition to the spectrum scarcity problem call for innovative approaches in addressing the resource management problem. In [16], a communication-theoretic model involving non-orthogonal sharing of RAN resources in uplink network slicing is studied with the three types of heterogeneous services such as eMBB, uRLLC, and mMTC, and the authors refer the approach to as H-NOMA. For details, see [17]; NOMA is an excellent choice for 5G services that have great spectrum efficiency (eMBB), comprehensive device connectivity (mMTC), and low transmission latency (uRLLC). The objective [18] is to simplify SIC while improving its value and utility for UE. In [19,20], a comprehensive survey of different candidate NOMA schemes for 5G is presented. In [21], rate-splitting multiple access (RSMA) is used for uRLLC transmission, where a uRLLC device separates its message into two sub-messages depending on the average signal-to-noise ratio (SNR) without immediate channel status information (CSI). The researchers investigated uRLLC and eMBB coexistence under a puncturing approach in multiple-input multiple-output (MIMO) NOMA systems by dividing the original issue into two sub-problems: user selection and power allocation in [22]. This article investigated the coexistence of eMBB and uRLLC services inside a cellular network with a reconfigurable intelligent surface (RIS) [23]. In [24], an enhanced Pre-emptive Scheduling (EPS) scheduler for joint URLLC, and eMBB traffic is proposed to extract the maximum possible eMBB ergodic capacity. In [25], eMBB and uRLLC communications were encoded in the cloud and cracked at the edge nodes to fulfil latency constraints in a Cloud radio access network (C-RAN) scenario. According to [26], eMBB transmission risk and uRLLC dependability are proposed as risk metrics for eMBB transmission to ensure a fair, proportionate allocation of resources to incoming uRLLC traffic. uRLLC scheduling is based on Deep Reinforcement Learning (DRL)-based learning to allocate traffic and optimize eMBB data rate and dependability [27]. This research uses linear, convex, and threshold eMBB rate loss to increase network resource usage and allocate the uRLLC traffic [28]. In [29], the authors overcome the co-channel problem of eMBB and uRLLC users by puncturing to improve the eMBB UE rate using a one-sided matching game. In [30], using a homogenous NOMA per slice in uplink and downlink with fairness enhances system sum-throughput for all users. The multi-connectivity (MC)-based strategy increases resources and minimizes wait times in [31] by spectrum sharing across various base stations and differential QoS for data and machine-to-machine (M2M) services in a dynamic network request.

There are many research efforts for better user association schemes suitable in NOMA for HetNets. In [32] power domain NOMA (PD-NOMA), game theory algorithms are proposed based on the QoS threshold to improve user association and power allocation. This work is presented by considering various case studies to demonstrate the effectiveness of PD-NOMA in ultra-dense networks. In [33], the unified PD-NOMA is utilized for user association in a dense heterogeneous network and improved user association, the overall system capacity, and throughput. In [34], the flexible user association is proposed in NOMA-based multiple base stations (BSs) networks to maximize the weighted sum rate of the system, and a user association optimization problem is formulated. While a NOMA user pairing scheme combined with the almost blank subframe (ABS) technology, and a dynamic NOMA power allocation scheme are presented to maximize the network fairness based on the optimized throughput of the edge users in [35]. But few writers focus on various services UEs’ associations with BSs. This study is aimed to improve isolation and flexibility by resolving the UE association in 5G [36]. In [37], using a matching game, UE association in a cellular virtualization network captures UE and BS preferences, QoS needs, and backhaul limitations. The Pointer Network (PtrNet) architecture implements NOMA’s joint UE pairing and association schemes [38]. Recently, a matching game has been proposed for wireless UE affiliation and resource allocation [39]. In [40], the authors intend to improve the eMBB network rate and decrease eMBB loss by convincing uRLLC UEs to cohabit with eMBB UEs by superposition readiness by matching game. In [41], matching game techniques for UE association in HetNets are presented, taking both downlink and uplink properties into account. Moreover, in [42], the optimization of downlink data rates was achieved with Radio access technology (RAT) heterogeneity by ensuring fairness and lowering uplink power usage. However, none of those mentioned works above considers intra and inter-isolation problems between slices when analyzing cell association and coexistence between uRLLC and eMBB slices.

### 1.2. Contribution

**Figure 1:**
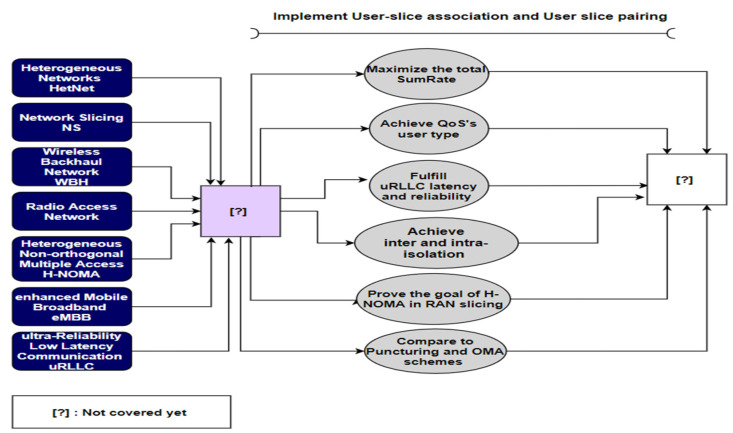
Contribution of this work (Table 1).

Utilizing the recent prosperity of using the matching theory to solve combinatorial optimization problem is proposed for eMBB UEs/uRLLC UEs association and pairing in HetNets in this paper. A scenario where eMBB and URLLC UEs with different requirements can be associated with different BSs and pair with each other is investigated. The main point of this paper can be summed up as follows in Figure 1:

o Joint user-association and pairing algorithms are considered for eMBB and uRLLC users in HetNets while considering the different association and pairing metrics required for each user type.
o In our system architecture, eMBB users are treated as downlink (DL) data-rate-hungry users and uRLLC users as latency and reliability-constrained users. The user-slice association and pairing processes are expressed as a multi-objective optimization problem to optimize the DL data rate for UEs and reduce the DL transmitted latency for uRLLC UEs whereas considering the users’ data rate-dependent QoS requirements and ensuring the intra-isolation for each slice by applying orthogonal frequency multiple access (OFDMA) technology between the similar service’s users; meanwhile, the inter-isolation between slices is assigned a DL threshold rate for each slice.
o We suggest a system model in which eMBB traffic is transmitted over long TTIs T, whereas uRLLC traffic is transmitted over short TTIs δ by superimposing the ongoing eMBB transmissions. Here, transmitting the incoming uRLLC traffic in the short TTI guarantees its delay demand. The data rate of eMBB traffic is picked up by Shannon’s capacity considering the effect of uRLLC transmissions, while uRLLC depends on the finite block-length capacity model due to its small packet size nature.
o We separate the multi-objective optimization problem into UE-slice association and UE-slice pairing sub-problems. Moreover, we improve a framework based on a one-to-many matching game to find a solution for the UE-slice association sub-problem in which BSs and UEs rate each other based on clear preference measures that define UEs’ and BSs’ needs. Meanwhile, the UE-slice pairing sub-problem is optimized by a one-to-one matching game in which the associated uRLLC and eMBB UEs with the same BS rank one another through some mini slots. To our knowledge, no matching game has been researched in HetNet to solve the eMBB UEs and uRLLC UEs association and pairing problem.
o We represent utility functions for UEs and BSs that consider the UEs’ different demands in terms of attained data rate, DL latency reliability, the threshold rate of each slice, dynamic criteria for limiting the number of attached UEs in HetNet, and the number of paired uRLLC UE with eMBB UEs (i.e., quota matching game), to achieve a reference rate for UEs.
o The joint UE-slice association and UE-slice pairing algorithms are proposed to solve this game. Then, the proposed algorithms are proven to arrive at a stable matching. As well as the computation complexity study for the suggested algorithms is given. Simulation results illustrate that our submitted algorithms outperform the comparable approaches, especially regarding DL latency performance for uRLLC UEs and DL rate performance for eMBB and uRLLC UEs. To illustrate the effectiveness of the proposed algorithms, we also compare their performance with other search schemes.

### 1.3. Organization

According to the following structure, the rest of this document is: Section 2 explains the models of the system. Section 3 explains the problem formulation, while Section 4 is performance analysis which shows a UE-slice association solution and UE-slice pairing solution. Section 5 is dedicated to numerical simulation and performance analysis. Furthermore, future work is discussed in Section 6. Finally, concluding remarks are presented in Section 7 The abbreviations used in this paper are given at the end.

**Table 1:** Notation List.

Notions	Definitions	Notions	Definitions
B	Set of base stations	$R_{u}^{min}$	Minimum DL required data rate for u∈U
L	Set of uRLLC users	$T_{l}^{max}$	Maximum DL tolerant delay on the system for l∈L
E	Set of eMBB users	$Z_{l}\left(t\right)$	payload size for l∈L
U	Set of eMBB and uRLLC users	$\lambda_{l}$	Arrival rate for l∈L
M	Number of mini slots in long TTI	$C_{bl}$	Block-length code for l∈L, b∈Ɓ
T	Duration of a RAN time slot	ε	uRLLC reliability probability
δ	Duration of a RAN mini slot	$V_{bl}^{nm}$	Channel dispersion for $l\in L,\;b\in Ɓ,n\in N_{b}^{1},m\in M$
S	Set of slices per BS	$T_{bl}^{WBH}$	WBH mean transmission packet delay for l∈L, b∈Ɓ
$I_{s}$	Set of users in each slice s∈S, BS b∈Ɓ	$T_{bl}^{RAN}$	RAN mean transmission packet delay for l∈L, b∈Ɓ
$N_{b}^{s}$	Set of virtual PRBs per BS’s slice s∈S,b∈Ɓ	${\mathrm{SIN}R}_{1b}$	Received SINR for a SBS from MBS b∈Ɓ, b≠1
$A^{U}$	Binary UE-slice association matrix	$\eta_{1b}$	Channel gain between MBS and SBS b≠1
$\alpha_{bu}^{nm}$	Power allocation coefficient for u∈U, b∈Ɓ	$o^{2}$	Noise power
$a_{bu}^{nm}$	Association decision variable, for $u\in U,\;b\in Ɓ,n\in N_{b}^{s},m\in M$	$SINR^{TH}$	Threshold SINR of WBH network
$X^{U}$	Binary Superposition matrix by H-NOMA	$br_{bu}$	Number of bits for a single successful WBH transmission
$x_{u\overset{`}{u}}^{nm}$	Superposition variable for l∈L, e∈E on $n\in N_{b}^{s}$ ,m∈M	$\tau^{WBH}$	WBH time slot
$\;Q_{bu}^{n}$	Integer number of mini slots for u∈U, b∈Ɓ	$W^{WBH}$	Bandwidth of the backhaul network
$y_{bu}^{nm}$	Received signal by user u∈U from BS b∈Ɓ $on\;n\in N_{b}^{s},m\in M$	${𝓆}_{bl}$	WBH required number of time slots l∈L, b∈Ɓ
$R_{sb}^{rsv}$	Reserved rate for s∈S, b∈Ɓ	$𝓅{𝓇}^{WBH}$	WBH Transmission success probability of one packet in a single transmission
${Ґ}_{bu}^{nm}$	Received SINR for UE u∈U from BS b∈Ɓ $\mathrm{on}\;n\in N_{b}^{s},m\in M$	ϱ	Pathloss exponent of WBH link
$\omega_{b}^{nm}$	Bandwidth of PRB $n\in N_{b}^{s}$ per m∈M	$T_{bl}^{tx}$	RAN transmission time l∈L, b∈Ɓ
$r_{bu}^{nm}$	Data rate for UE u∈U from BS b∈Ɓ on $n\in N_{b}^{s}$, per m∈M	$\rho_{bl}$	Number of paired mini slots for l∈L with e∈E
$R_{bu}$	Overall DL received rate for u∈U from BS b∈Ɓ	$D_{l}$	maximum threshold RAN’s packet delay
$\Omega_{bu}^{nm}$	PRBs allocation decision variable, for u∈U, b∈Ɓ,m∈M	$T_{bl}^{RTT}$	HARQ retransmission delay l∈L, b∈Ɓ
$\gamma_{bu}^{m}$	Number of allocated PRBs for u∈U, b∈Ɓ,m∈M	$T_{bl}^{WT}$	Superimposed time for l∈L pairing with eMBB UE e∈E
$f_{bu}^{m}$	Number of minimum required PRBs for u∈U, b∈Ɓ,m∈M	${\bar{T}}_{bl}$	Total mean transmission packet delay on the system for l∈L, b∈Ɓ

## 2. System Model

### 2.1. Network Model and Assumptions

**Figure 2:**
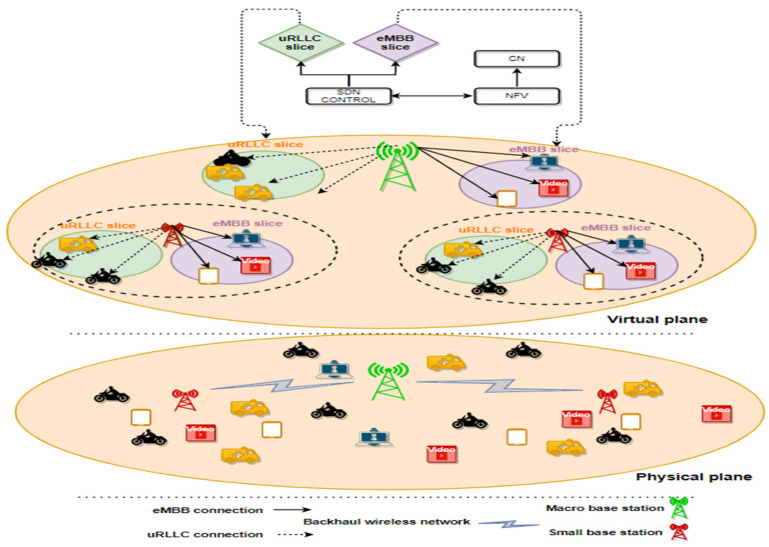
System Model.

We consider a two-tier downlink H-NOMA cellular network which consists of wireless backhaul and radio access networks with a single infrastructure provider (InP), where 1st tier consists of an MBS while 2nd tier models SBSs. The set of BSs is denoted as $b=\left\{1,\;2,\;..,\ldots \;B\right\}$ when *b* = 1 is a MBS. However, *b* ≠ 1 is a SBS. We assume that the out-band operation mode is operated on the HetNet system. Thus, non-line-of-sight (NLOS) backhaul and access links are operated on different carriers; thus, separation in the frequency domain is provided to achieve efficient spectrum usage [43,44]. Also, we assume that all BSs utilize the same frequency band [45], resulting in SBSs being underlined by spectrum sharing with one MBS. Let $l=\left\{l_{1},\;l_{2},\;l_{3},\;\ldots ,\;L\right\}\;$and$\;e=\left\{e_{1},\;e_{2},\;e_{3},\;\ldots ,\;E\right\}$ denote the set of uRLLC and eMBB UEs, respectively, are randomly distributed over the entire network area. The set of all UEs containing uRLLC UEs and eMBB UEs is represented by U *=* L U E, where the number of eMBB UEs is represented by $u\in U=\left\{1,\;2,\ldots ,k\right\}$ and the set of uRLLC UEs are indicated by $u=\left\{k+1,\;\ldots ,\;U\right\}.$ We assume the total transmitted power’s MBS is equally distributed between WBH and RAN as $P_{1b}$ where b ϵ Ɓ, b≠1 and $P_{1}^{N}$,respectively. Each BS will have $N_{b}$ physical resource blocks (PRBs) in RAN, distributed amongst its attached users depending on the available system band. We suppose that the overall transmitted power of each BS’s RAN is allocated evenly over the available PRB ($P_{b}^{n}$). In this system design, we slice and assign PRBs as virtual resources into two RAN slices to serve uRLLC, and eMBB users, as illustrated in Figure 2. The set of all slices is denoted by $s\in S=\left\{1,\;2\right\},$ where s=1 refers to the uRLLC slice and s=2 is the eMBB slice. Each slice s has its own set of users denoted by $I_{s}$, let ı = $\cup_{s}^{S}{Ι}_{s}$ where | · | is the cardinality of the set of all UEs. Considering non-orthogonal slicing [36], all virtual PRBs are used for uRLLC and eMBB transmissions at each slice’s BS as $N_{b}^{1}=N_{b}^{2}=N_{b}$ ∀ b∈Ɓ, respectively. Since the connection and pairing processes are occurred on a larger time scale than the channel change, the small-scale fading term swings fast enough to be averaged in the measurements of its channel during the connection time.

### 2.2. Joint User Pairing and Association Matrix

Let us know a binary UE association matrix as $A^{U}\in {\left\{0,1\right\}}^{B\times U\times N_{B}^{S}\times M}$, where $a_{bu}^{nm}$ = 1 if a UE u is only associated with one base station and assigned to a PRB n on mini-slot m, and $a_{bu}^{nm}=0$, otherwise. Let us express a binary UE coupling matrix $X^{U}\in {\left\{0,1\right\}}^{U\times U^{\prime }\times N_{B}^{S}\times M}$, where $x_{u\overset{`}{u}}^{nm}=1$ if an eMBB UE u is paired with an uRLLC UE $\overset{`}{u}$ by superposition scheme, at the same BS, and $x_{u\overset{`}{u\;}}^{nm}=0$, otherwise. $\;Q_{bu}^{n}$ denotes the integer number of mini slots assigned to UE u when attached with BS b; if a UE is eMBB then $\;Q_{bu}^{n}=M$ (along TTI T) as for a time slot; but if a UE is uRLLC so$\;Q_{bu}^{n}$ is based on the paired eMBB UE.

To formulate the user association, we define the association matrix $A^{U}$ as

(1) $$a_{bu}^{nm}=\left\{\begin{array}{c} 1,\;\;\;\;\mathrm{if}\;\mathrm{UE}\;u\;\mathrm{is}\;\mathrm{associated}\;\mathrm{with}\;\mathrm{BS}\;b\;\\ 0,\;\;\;\;\;\;\;\;\;\;\;\;\;\;\;\;\;\;\;\;\;\;\;\;\;\;\;\;\;\;\;\;\;\;\;\;\;\;\;\;\;\;\;\;\;\;\;\mathrm{otherwise}. \end{array}\right.$$

The adoption of PD-NOMA scheme by the user u is represented by

(2) $$x_{u\overset{`}{u}}^{nm}=\left\{\begin{array}{c} 1,\;\;\mathrm{if}\;\mathrm{user}\;u\;\mathrm{is}\;\mathrm{superposited}\;\mathrm{with}\;\overset{`}{u}\mathrm{are}\;\mathrm{associated}\;\mathrm{with}\;\mathrm{the}\;\mathrm{same}\;\mathrm{BS}\;,\\ 0,\;\;\;\;\;\;\;\;\;\;\;\;\;\;\;\;\;\;\;\;\;\;\;\;\;\;\;\;\;\;\;\;\;\;\;\;\;\;\;\;\;\;\;\;\;\;\;\;\;\;\;\;\;\;\;\;\;\;\;\;\;\;\;\;\;\;\;\;\;\;\;\;\;\;\;\;\;\;\;\;\;\;\;\;\;\;\;\;\;\;\;\;\;\;\;\;\;\;\;\;\;\;\;\;\;\;\;\;\mathrm{otherwise}. \end{array}\right.$$

### 2.3. Signalling Model

**Figure 3:**
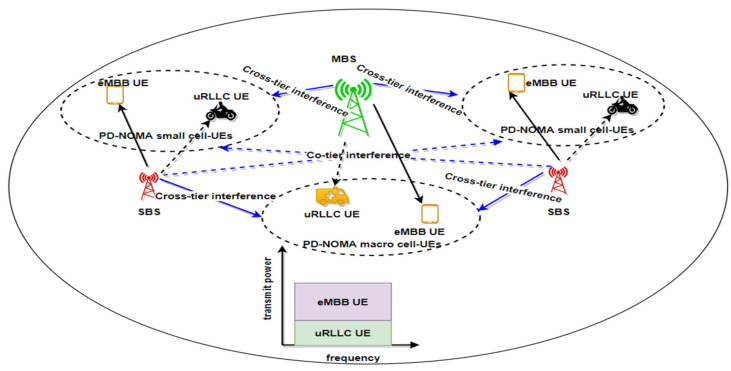
An illustration of eMBB and uRLLC users down-link PD-NOMA HetNet scheme.

Two types of interferences should be considered in the proposed multi-cell H-NOMA system. Firstly, the inter-BS interference is generated by the signals transmitted from the un-associated BSs. Secondly, the intra-BS interference is produced by the co-channel interference of H-NOMA schemes. In any coalition, the received signal at user u is shown as follows [33,34] in Figure 3:

(3) $$y_{bu}^{nm}=\underset{\underset{\underset{\left(\mathrm{Unwanted}\;\mathrm{Signal}\right)}{\mathrm{inter}-\mathrm{BS}\;\mathrm{interference}}}{⏟}}{\sum_{h\in Ɓ\backslash \{b\}}\sum_{\overset{`}{u}\in \{k+1,\ldots ,U\}\backslash \{u\}}\sqrt{\alpha_{hu}^{nm}\;P_{h}^{n}}{ℊ}_{hu}^{nm}x_{u\overset{`}{u}}^{nm}{𝓇}_{hu}^{nm}}+\underset{\underset{\underset{\left(\mathrm{Unwanted}\;\mathrm{Signal}:\;\mathrm{Remove}\;\mathrm{using}\mathrm{SIC}\right)}{\mathrm{intra}-\mathrm{BS}\;\mathrm{interference}}}{⏟}}{\sum_{b\in Ɓ}\sum_{u,\overset{`}{u}\in U}\sqrt{(1-\alpha_{bu}^{nm})P_{b}^{n}}{ℊ}_{bu}^{nm}x_{u\overset{`}{u}}^{nm}}{𝓇}_{b\overset{`}{u}}^{nm}\;+\underset{\underset{\mathrm{Wanted}\;\mathrm{Signal}\;}{⏟}}{\sqrt{\alpha_{bu}^{nm}\;P_{b}^{n}}{ℊ}_{bu}^{nm}x_{u\overset{`}{u}}^{nm}{𝓇}_{bu}^{nm}}+o^{2}$$

where $Ɓ\backslash \left\{b\right\}$ is the collection of interfering nodes that utilise the same frequency channel, ${𝓇}_{bu}^{nm}$ the signal transmitted from the BS to the u UE, $\alpha_{bu}^{nm}\in \left[0,\;1\right]$ denotes the power allocation coefficient for the UE u associated with BS b on PRB n; $P_{b}^{n}$ denotes the transmit power of BS b over PRB n to UE u; ${ℊ}_{bu}^{nm}$ represents the channel gain between BS b and UE u at mini slot m and $(1-\alpha_{bu}^{nm})\;P_{b}^{n}{ℊ}_{bu}^{nm}$ denote the received interference from the coexisting another user. Moreover, let ${ℊ}_{u}$ $=({ℊ}_{1u}$, ${ℊ}_{2u},\;$…, ${ℊ}_{Ɓu})$ ∈G with the perfect CSI. $o^{2}$ is the additive noise power.

When H-NOMA is applied, each PRB can mostly serve two different services UEs. Implementing H-NOMA brings more sophisticated co-channel interferences (CCI) to the existing networks; to optimize the main problem without CCI employing perfect SIC, both eMBB and uRLLC UEs receive the superimposed signal, and each UE executes SIC to decode its message [46]. We assume the stronger user is a uRLLC UE which only can decode and remove CCI from the weak user is eMBB UE. While the eMBB UE with weaker channel conditions cannot wholly eliminate the interference of uRLLC UEs’ signals, which results in maximized throughput for the eMBB UE with contrast [40] and prove it with the results in Section 5 Because our approach is based on uRLLC UE is superimposed with eMBB UE through a few mini slots and assigned high power for eMBB UE to reduce the latency by providing a high rate to the paired uRLLC UEs at a mini slot and achieving high reliability. Thus, the effect of CCI on eMBB UE is less than if the conventional pairing is along a TTI.

As well as, to guarantee inter-slice interference isolation between slices and ensure the QoS for each UE-slice according to H-NOMA technology, we assume $R_{sb}^{rsv}$ is the reserved rate for each slice $s\in S=\left\{1,\;2\right\}$ in each BS [47,48]. The total rate of the cell is denoted as $R_{b}^{rsv}$=$R_{1b}^{rsv}+$ $R_{2b}^{rsv}$; based on the average downlink physical data rate of LTE system is achieved by [49].

Under the superposition scheme, the SINR ${Ґ}_{bu}^{nm}$ is received by the UE u∈U from a BS b∈Ɓ on a PRB $n\in N_{b}^{s}$ per mini slot m∈M as, can be as follows

(4) $${Ґ}_{bu}^{nm}=\frac{\alpha_{bu}^{nm}\;P_{b\;}^{n}{ℊ}_{bu}^{nm}\;}{(1-\alpha_{bu}^{nm})\;P_{b}^{n}{ℊ}_{bu}^{nm}+\sum_{h\in Ɓ\backslash \left\{b\right\}}\alpha_{hu}^{nm}\;P_{hu\;}^{n}{ℊ}_{hu}^{nm}+o^{2}},$$

### 2.4. Data Rate Expression Based on Shannon Capacity Model

The DL achievable data rate of a UE u attached with BS b with bandwidth $\omega_{b}^{nm}$ over one PRB n per mini slot m can be calculated as,

(5) $$r_{bu}^{nm}=\omega_{b}^{nm}\log_{2}(1+{Ґ}_{bu}^{nm}),$$

We denote the total received rate of a UE u, represented by $R_{bu}$, calculated as follows,

(6) $$R_{bu}=\sum_{\;b\in Ɓ}\sum_{\;u,\overset{`}{u}\;\in U}a_{bu}^{nm}x_{u,\overset{`}{u}}^{nm}\;\;Q_{bu}^{n}\;\Omega_{bu}^{nm}\;\gamma_{bu}^{m}\;r_{bu}^{nm},$$

$\Omega_{bu}^{nm}$ is the allocation indicator of the PRB n to UE u in BS b per mini slot m, where $\Omega_{bu}^{nm}$ = 1 if a PRB n is allocated to a UE u and $\Omega_{bu}^{nm}$ = 0, otherwise. Furthermore, $\gamma_{bu}^{m}$ is the fraction of the PRB allocated to UE u and is calculated as $\gamma_{bu}^{m}=\lceil \frac{R_{bu}}{r_{bu}^{nm}}\rceil$, where ⌈·⌉ is the ceiling function.

(7) $$\gamma_{bu}^{m}\geq \;f_{bu}^{m},$$

When a UE u is associated with a BS b, $\gamma_{bu}^{m}$ PRBs are allocated to a UE u, and this BS is duty to achieve the following condition

(8) $$R_{bu}\geq \;R_{u}^{min},$$

### 2.5. uRLLC Traffic

The uRLLC UEs are with delay-sensitive machine-type traffic and require high transmission reliability. Therefore, a UE l associates with a base station and superposes with an eMBB UE e who satisfy its required QoS constraint $T_{l}^{max}$ & $R_{l}^{min}$.

#### 2.5.1. uRLLC Data Rate Depending on Finite Block-Length Coding

The uRLLC’s bursts of small payload sizes of $Z_{l}\left(t\right)$ bytes arrive at the network according to a Poisson Point Process (PPP) with an arrival rate of $\lambda_{l}$ [payload/s] [50]. Due to the small packet size of uRLLC traffic, Shannon’s data rate formulation cannot be used directly. Consequently, the DL data rate of uRLLC UE in (5) is edited based on [50] i.e.,

(9) $$r_{bl}^{nm}=\omega_{b}^{nm}\log_{2}(1+{Ґ}_{bl}^{nm})-\sqrt{\frac{V_{bl}^{nm}}{C_{bl}}}\;Q^{-1}\left(\varepsilon \right),$$

where $C_{bl}$ is the block-length code, and $Q^{-1}\left(\varepsilon \right)\;$is the inverse of the complementary Gaussian cumulative distribution Q-function with the probability of decoding error ε and $V_{bl}^{nm}$ is the channel dispersion which is represented by

(10) $$V_{bl}^{nm}=1-\frac{1}{1+\left(1+{Ґ}_{bl}^{nm}\right)},$$

#### 2.5.2. uRLLC Mean Packet Delay

In this system, the deadline interval of uRLLC UE l associated with BS b is contributed by the time processing of backhaul and RAN, are represented as $T_{bl}^{WBH}$ and $T_{bl}^{RAN}$, respectively.

#### 2.5.3. Wireless Backhaul Network

The analysis for backhaul connection behind the core gateway (GW) is neglected since it is common for all BSs and is assumed to be ideal. Thus, the received signal-to-interference-plus-noise ratio (SINR) to the $b^{th}$ SBS from MBS on WBH is displayed as,

(11) $${\mathrm{SIN}R}_{1b}=\frac{P_{1b}\;\eta_{1b}}{\sum_{h\in Ɓ\backslash \left\{b\right\}}P_{1h}\;\eta_{1h}+o^{2}},$$

where $\eta_{1b}$ represents the channel gain between MBS and SBS. For the WBH network, the transmission succeeds if the received SINR is ${\mathrm{SIN}R}_{1b}\leq SINR^{TH}$, $SINR^{TH}$ is a threshold SINR. Otherwise, the transmission has failed, necessitating retransmission. Clearly, the transmission success probability in a single transmission attempt is contingent upon the link length, bandwidth, and time slot length. The wireless backhaul transmission is time-slotted, and one packet is transmitted in each time slot. One hop is assumed for sub-6 GHz backhaul since the distance between the SBS and the MBS is often not great [51,52]. Meanwhile, the radio access network connects users with (macro cell or small cell) BSs through wireless links, which usually have only one hop. Considering the Shannon capacity formula, where the number of bits $br_{bl}$ that can be transmitted from BS b to uRLLC UE l in a single successful transmission through backhaul time slot $\tau^{WBH}$ and $\;W^{WBH}$ backhaul system bandwidth,

(12) $$br_{bl}=\tau^{WBH}\;W^{WBH}\log_{2}(1+SINR^{TH}),$$

Thus, $T_{bl}^{WBH}$ the mean transmission packet delay for uRLLC UE l over the sub-6 GHz WBH link, is based on $SINR_{1b}$ in (11), can be expressed as follows,

(13) $$T_{bl}^{WBH}=\tau^{WBH}\;{𝓆}_{bl}\frac{1}{𝓅{𝓇}^{WBH}}=\tau^{WBH}\;{𝓆}_{bl}\;\frac{2}{\beta \;\sin\left(2\pi \beta \right)\;{\left(SINR^{TH}\right)}^{2\beta }},$$

where ${𝓆}_{bl}$ is the required number of WBH time slots of a uRLLC UE for delivering its packet and equals $\left\lceil \frac{Z_{l}\left(t\right)}{br_{bl}}\right\rceil$; $𝓅{𝓇}^{WBH}$ is the transmission success probability in a single transmission attempt to calculate the average number of transfers required to deliver a packet successfully as $\frac{1}{𝓅{𝓇}^{WBH}}$, and β=2ϱ, ϱ>2 *is* pathloss exponent of WBH link.

#### 2.5.4. Wireless RAN Network

$T_{bl}^{RAN}$ consists of BS process time, UE process time, frame alignment time, queue time, and transmission time [31,50,53]. In this work, uRLLC and eMBB traffic are independent in slices’ various services, so the queue of eMBB does not affect uRLLC. The system always has resources to service the uRLLC immediately as it arrives, which leads to the uRLLC UE having no queueing delay. BS and UE process time are bound by three OFDM symbol durations, which are very small and refer to the equipment computing capacity. As for, frame alignment time is upper bounded by the short TTI interval (δ), and transmission time $T_{bl}^{tx}$ is related to the data rate of UE l. In superposition operation, UE l is paired with eMBB e, if its load $R_{l}^{min}$ needs to be transmitted within the stipulated period δ, $T_{bl}^{tx}=\rho_{bl}\;\delta$, where $\rho_{bl}$ is the number of mini slots in which uRLLC UE l pairs with eMBB UE e and the following condition is satisfied, $\underset{l\in L}{\max}\frac{R_{l}^{min}}{R_{bl}T_{bl}^{tx}}\leq D_{l},$ where$\;D_{l}$ indicates the maximum RAN packet delay threshold of uRLLC UE l and $R_{bl}$= $r_{bl}^{nm}*\gamma_{be}^{m}$. In case of failure, the packet is subject to additional retransmission delay $T_{bl}^{RTT}$ is a round trip delay of a HARQ retransmission until either it is decoded successfully, or the maximum number of retransmissions is reached. This work considers uRLLC UE u∈U scheduled with short TTI units composed of two OFDM symbols and Short HARQ RTT. Thus, we can determine the mean transmission packet delay $T_{bl}^{RAN}$ of a uRLLC UE is as follows [50,53,54],

(14) $$T_{bl}^{RAN}≅\;T_{bl}^{tx}+T_{bl}^{WT}+T_{bl}^{RTT},$$

where $T_{bl}^{WT}$ is the superimposed mini-slot time for uRLLC UE l to pair with eMBB UE e. Each eMBB UE may be paired with more than one uRLLC UE, so the eMBB TTI is split into mini-slots; each paired uRLLC UE takes several $\rho_{bl}\;$mini-slots, and one mini slot is spaced to avoid intra-slice interference between uRLLC UEs that are paired.

To compute the average DL packet transmission delay of this system for uRLLC UE l [53,54], i.e.,

(15) $${\bar{T}}_{bl}=\left\{\begin{array}{c} T_{bl}^{RAN},\;\;\;\;\;\;\;\;\;\;\;\;\;\;\;\;\;\;\;\;\;\mathrm{if}\;b=1\\ T_{bl}^{WBH}+T_{bl}^{RAN},\;\;\;\;\;\;\mathrm{if}\;b\neq 1 \end{array}\right.,$$

On the other side, the reliability of uRLLC can be improved by making sure that its outage probability is less than a certain threshold, according to [27],

(16) $$Pr\;(\sum_{l\in \;L}R_{bl}<a_{bl}^{nm}R_{l}^{min})\leq \varepsilon ,$$

If a uRLLC UE l connects with BS b and paires with an eMBB UE e, it is the duty of this BS and eMBB UE to achieve its QoS. i.e.,

(17) $$R_{bl}\geq \;R_{l}^{min}\;\&\&\;\;{\bar{T}}_{bl}\;\leq T_{l}^{max},$$

## 3. Problem Formulation

An association and pairing approach is now addressed by jointly considering eMBB and uRLLC UEs’ different demands. Since the uRLLC UEs should operate on a constrained received latency, DL latency should be considered during the connection process for uRLLC users. In contrast to uRLLC UEs’ needs, eMBB communications typically demand a high DL data rate. Thus, the DL data rate is a crucial association measure for eMBB users. Therefore, the UE-slice association and the UE-slice pairing indicators can be designed to maximize the overall DL data rate for eMBB UEs and minimize the DL transmitted latency for uRLLC UEs. Thus, the user-slice association (U−S. A) and the UE-slice pairing (U−S. P) problem can be represented as,

(18) $$O.P.T1:\underset{\left\{a,x\right\}}{\max}\;{\left[F_{1},-F_{2}\right]}^{T},$$

It can be observed that the O.P.T1 (18) is a nonconvex mixed-integer programming problem, where the ideal solution is challenging to obtain. The subsequent subsection will convert the problem into a pure combinatorial optimization issue. Therefore, we adopt a suboptimal method and decompose the O.P.T1 into two designed sub-problems, as described in the next sub-sections. The first sub-problem is to create the user-slice association decision matrix A. The second sub-problem is to create the user-slice pairing decision matrix X. Thus, we suggest solving this problem using two-sided matching, as seen in the next section.

### 3.1. UE-Slice Association Sub-Problem

First, we concentrate on the association of users to BSs to boost overall DL data rates and minimize uRLLC latency while considering the RAN capacity for each BS and guaranteeing the intra-slice and inter-slice isolation. The U-S. A solution can be obtained by solving the following optimization sub-problem:

(19) $$O.P.T1-U-S.\;A:\underset{\left\{a\right\}}{\max}\;{\left[F_{1}\left(a\right),-F_{2}\left(a\right)\right]}^{T},$$

S.t       (8), (16), (17),(20)

(21) $$\sum_{u\in U}a_{bu}^{nm}\gamma_{bu}^{m}\leq N_{b}^{s}\;\;\;\;\;\forall \;m\in M,\;s\in S,\;b\in B,$$

(22) $$\sum_{u\in U}a_{bu}^{nm}R_{bu}\leq R_{sb}^{rsv}\;\;\;\;\;\forall \;m\in M,\;s\in S,\;b\in B$$

(23) $$\sum_{,\;b\in B}a_{bu}^{nm}=1\;\;\forall \;m\in M,\;s\in S,\;u\in U$$

(24) $$\sum_{,n\in N_{b}^{s}}\Omega_{bu}^{nm}\leq 1\;\;\;\;\;\forall \;m\in M,\;s\in S,\;u\in U,\;b\in B$$

(25) $$a_{bu}^{nm}\in \left\{0,1\right\}\;\;\;\;\;\forall \;m\in M,n\in N_{b}^{s},\;\;s\in S,\;u\in U,\;b\in B$$

(26) $$f_{bu}^{m}\in \left\{1,2,\ldots ,N_{b}^{s}\right\}\;\;\;\;\;\forall \;m\in M,\;s\in S,\;u\in U,\;b\in B$$

where $F_{1}\left(a\right)=\sum_{b\in Ɓ}\sum_{u\in U}\sum_{s\in S}\sum_{n\in N_{b}^{s}}\;a_{bu}^{nm}\;R_{bu}$, that is, the overall data rate for eMBB and uRLLC UEs, and $F_{2}\left(a\right)=\sum_{b\in B}\sum_{l\in L}a_{bu}^{nm}\;{\bar{T}}_{bl}$, that is, the overall mean transmitted packet latency for uRLLC UEs. Note that to reduce the goal using the minus sign in (19), initially, we neglect the superimposed time $T_{bl}^{WT}=0$. Constraint (21) allows the attached UE to utilize the PRBs without bypassing the base station resource harvest. Condition (22) ensures the inter-slice interference isolation in each BS. Constraint (23) ensures that each UE is associated with one BS or not attached at most, while condition (24) specifies that each PRB per TTI is allocated to one UE is in the same service’s slice to achieve intra-slice isolation. Condition (25) indicates that the connection indicators have possible binary values, as for (26) indicates that it should occupy integer values and not exceed the maximum number of PRBs at each slice’s BS.

Therefore, we observed that the O.P.T1−U−S. A is a non-linear objective and complicated sub-problem due to the discrete features of the association indices. To solve the O.P.T1−U−S. A sub-problem, we develop an algorithm for UE-slice association, in which the one-to-many matching game [36,37,39] is used, as described in Section 4.1.

### 3.2. UE-Slice Pairing Sub-Problem

Then, the optimum pairing for uRLLC UEs and eMBB UEs is achieved to completely maximize the network’s total throughput and maintain the QoS requirements for users using the superposition (H-NOMA) scheme between eMBB and uRLLC UEs, additionally considering the intra-slice and inter-slice isolation and the limited capacity for the service’s users, whereas we consider only the eMBB and uRLLC users are associated with the same BS and consider the same objective of (19) with fixed $a^{t}$, ∀ T. Therefore, the (U−S. P) sub-problem can be designed as follows:

(27) $$O.P.T1-U-S.\;P:\underset{\left\{x\right\}}{\max}\;{\left[F_{1}\left(x\right),-F_{2}\left(x\right)\right]}^{T},$$

S.t      (8), (16), (17)(28)

(29) $$x_{u\overset{`}{u}}^{nm}\in \left\{0,1\right\}\;\;\;\;\;\forall \;m\in M,\;n\in N_{b}^{s},\;\;s\in S,\;u\in L,\overset{`}{u}\in E,\;b\in B$$

(30) $$T_{bl}^{WT}\in \left\{1,2,\ldots ,M\;\right\}\;\;\;\;\;\forall \;l\in L,\;b\in B$$

where $F_{1}\left(x\right)=\sum_{b\in Ɓ}\sum_{u,\overset{`}{u}\in U}\sum_{s\in S}\sum_{n\in N_{b}^{s}}\;x_{u\overset{`}{u}}^{nm}\;R_{bu}$, and $F_{2}\left(x\right)=\sum_{b\in B}\sum_{l\in L}x_{u\overset{`}{u\;}}^{nm}\;{\bar{T}}_{bl}$. Condition (28) means that the pairing indicators values are binary values. In this case, constraint (29) should take into consideration the superimposed time of pairing in our calculations, which is a random integer mini-slot time. Therefore, a one-to-one matching game [40,55,55] is used to acquire the UE-slice pairing solution, which is explained in Section 4.2.

## 4. Solution Using Matching Game-Based U-S. A and U-S. P Algorithms

In this section, we propose two matching algorithms to solve the U-S. A and U-S. P formulated sub-problems. Since O.P.T1−U−S. A and O.P.T1−U−S. P sub-problems are formulated as a one-to-many and one-to-one matching game, respectively. The matching function of U−S. A is defined by $\mu_{U-S.\;A}$ with a tuple $\left(B,U,\Theta ,{≻}_{B},{≻}_{U}\right)$ and the U−S. P is represented as $\mu_{U-S.\;P}$ with a tuple $\left(L,E,\tilde{\Theta },{≻}_{L},{≻}_{E}\right).$ In the U−S. A matching function, ${≻}_{B}$=${\left\{{≻}_{b}\right\}}_{b\in B\;}$ and ${≻}_{U}$= ${\left\{{≻}_{u}\right\}}_{u\in U\;}$ indicates the preference relations of the BSs and UEs players, respectively. As for the U−S. P matching function, ${≻}_{L}={\left\{{≻}_{l}\right\}}_{l\in L\;}$ and ${≻}_{E}={\left\{{≻}_{e}\right\}}_{e\in E\;}$ denote the preference relations of the associated uRLLC UEs and eMBB UEs with the same BS, respectively. Assume that Θ and $\tilde{\Theta }$ are the quota for O.P.T1−U−S. A and O.P.T1−U−S. P, respectively.

### 4.1. One-Sided Matching Based Solution Approach Sub-Problem (19)

There are diverse preferences utilities that can be supplied for players depending on their tendencies. For O.P.T1−U−S. A, we erect the preference relations of UEs (eMBB and uRLLC UEs) and BSs for selecting the best match according to the utility functions listed as $\Psi_{u}\left(b\right)$ and $\Psi_{b}\left(u\right)$.

#### 4.1.1. UEs Utility Function

Since eMBB UEs are data-rate-hungry, we utilize the DL attainable data rate as the utility function $\Psi_{e}\left(b\right)$, which can be expressed as follows:

(31) $$\Psi_{e}\left(b\right)=R_{be},$$

Meanwhile, uRLLC UEs are deemed urgent-latency UEs; thus, the utility attained by an uRLLC UE when it is associated with the BS is introduced as a function of the DL transmitted latency and reliability as follows:

(32) $$\Psi_{l}^{1}\left(b\right)=R_{bl}\;\&\;\Psi_{l}^{2}\left(b\right)={\bar{T}}_{bl},$$

#### 4.1.2. Base Stations Utility Function

The base station’s utility function must be efficient for the user-slice association. Where each BS wants to serve UEs with the highest rate to optimize the overall DL throughput based on the following utility function:

(33) $$\Psi_{b}\left(u\right)=R_{bu},$$

The major details of the U−S. A algorithm are depicted in Algorithm 1. After initialization, each UE erects its preference relations $H_{u}$ ${≻}_{u}$ based on (31) and (32) (step 4). Similarly, the preference list for each BS $H_{b}$ is constructed based on the preference utility (23). At each iteration It, all unassigned UEs (eMBB and uRLLC UEs) send attachment requests to their preferred BSs (steps 5–9). Each BS will then determine whether to accept the requests or not based on its defined utility and quota $\Theta_{b}$ ($\Theta_{b}^{rem}$ and $\Theta_{b}^{th}$ available radio resources and rate for each slice of a BS) that restricts the number of attached UEs to avert Quality of Experience (QoE) retrogression. If a BS has sufficient $\Theta_{b}^{rem}$ and $\Theta_{b}^{th}$ to accept, it accepts the request and updates $\Theta_{b}^{rem}$, $\Theta_{b}^{th}$ and $\mu_{U-S.\;A}^{\left(It\right)}\left(b\right)$ (steps 10–14). Otherwise, if the quota is not sufficient, but the utility (31)/(32) of the requesting UE u is more significant than a UE $\hat{u}$ that was accepted in the previous round, the BS b determines all its current matches $\hat{u}$ which have a worse ranking than according to steps $H_{b}^{It}$ (steps 15–17). Each least preferred $\hat{u}$ is then sequentially removed, and $\Theta_{b}^{rem}$, $\Theta_{b}^{th}$, $\mu_{U-S.\;A}^{\left(It\right)}\left(b\right)$ and the minimum preference list ($\xi_{mp}$) is updated until it can be accepted or there is no more to reject (steps 18–29). After rejecting all UEs $\hat{u}$ and the available PRBs at BS are still insufficient to admit a UE u. Thus, it is refused and assigned to $\xi_{mp}$ (steps 30–31), i.e., the refused UEs will send an attachment request in the next round to the next BS in their preference lists. Consequently, BS b removes $\xi_{mp}$ its less preferred UEs from the respective lists $H_{b}$. Similarly, these UEs remove a BS b from their preference lists (lines 32–33). After many iterations It, there are no more bidding UEs; thus, the algorithm converges to a stable match.
**Algorithm 1:** Matching Game for User-Slice Association1:  **Input:**
U, B
2:  **Output:** Find stable Matching $\;\mu_{U-S.\;\;A}^{\left(It\right)}$
3:  **Initialization:**
It=0,$\;\mu_{U-S.\;\;A}^{\left(It\right)}={\left\{\mu_{U-S.\;\;A}^{\left(It\right)}\left(b\right),\;\mu_{U-S.\;\;A\;}^{\left(It\right)}\left(u\right)\right\}}_{\forall u\in U,b\in B}=\emptyset ,\;\;\xi_{mp}=\emptyset ,\;z_{b}^{\left(It\right)}=\emptyset ,\;\Theta_{b}=\left\{\Theta_{b}^{rem}=N_{b}^{s},\;\;\Theta_{b}^{th}=0\right\}{\;}_{s\in S,\;b\in B}$
4:  Every UE construct ${≻}_{u}$ using $\Psi_{u}\left(b\right)$
5:  **Repeat**6:   
It←It+1
7:    **for**
 b∈B
**do**8:     **for**
 u∈U do with b as its best preferred in $H_{u}^{It}$
**do**9:      **while**
$u\notin \mu_{U-S.\;\;A}^{\left(It\right)}\left(b\right)$ and $H_{u}^{It}\neq \emptyset$
**do**10:        **If**
$N_{b}^{s}\geq f_{bu}^{m}$ and $R_{sb}^{rsv}\geq R_{bu}$
**then**11:        **If**
$\Theta_{b}^{rem}\geq f_{bu}^{m}$ and $\Theta_{b}^{th}<R_{sb}^{rsv}$
**then**
12:          
$\mu_{U-S.\;\;A}^{\left(It\right)}\left(b\right)\leftarrow \mu_{U-S.\;\;A}^{\left(It\right)}\left(b\right)\cup \left\{u\right\},\;\;$
13:          
$\Theta_{b}^{rem}\leftarrow \Theta_{b}^{rem}-f_{bu}^{m},\;$
14:          
$\Theta_{b}^{th}\leftarrow \Theta_{b}^{th}+R_{bu}$
15:        **else**
16:          
${\hat{H}}_{b}^{It}\leftarrow \left\{\hat{u}\in \mu_{U-S.\;\;A}^{\left(It\right)}\left(b\right)|u{≻}_{b}\hat{u}\right\},$
17:          $\xi_{mp}$ $\leftarrow \hat{u}\in {\hat{H}}_{b}^{It}$18:          **while**
${\hat{H}}_{b}^{It}\cup$ ($\Theta_{b}^{rem}<f_{bu}^{m}$) **do**19:            $\mu_{U-S.\;\;A}^{\left(It\right)}\left(b\right)\leftarrow \mu_{U-S.\;\;A}^{\left(It\right)}\left(b\right)\backslash \left\{\xi_{mp}\right\}$,21:            ${\hat{H}}_{b}^{It}\leftarrow {\hat{H}}_{b}^{It}\backslash \left\{\xi_{mp}\right\}$,22:            
$\Theta_{b}^{rem}\leftarrow \Theta_{b}^{rem}-f_{bu}^{m}$
23:            
$\Theta_{b}^{th}\leftarrow \Theta_{b}^{th}+R_{bu},$
24:            $\xi_{mp}$ $\leftarrow \hat{u}\in {\hat{H}}_{b}^{It}$25:            **If**
$\Theta_{b}^{rem}\geq f_{bu}^{m}$ and $\Theta_{b}^{th}<R_{sb}^{rsv}\;$**then**
26:             $\mu_{U-S.\;\;A}^{\left(It\right)}\left(b\right)\leftarrow \mu_{U-S.\;\;A}^{\left(It\right)}\left(b\right)\cup \left\{u\right\},\;\;$
27:             $\Theta_{b}^{rem}\leftarrow \Theta_{b}^{rem}-f_{bu}^{m},\;$
28:             $\Theta_{b}^{th}\leftarrow \Theta_{b}^{th}+R_{bu}$
29:            **else**
30:              $\xi_{mp}$ ←u,31:              $z_{b}^{\left(It\right)}=\left\{z\in H_{b}^{It}|\;\xi_{mp}{≻}_{b}z\right\}\cup \left\{\xi_{mp}\right\}$
32:              **for**
$z\in z_{b}^{\left(It\right)}$
**do**33:                $H_{z}^{It}\in H_{z}^{It}\backslash \left\{b\right\}$, $H_{b}^{It}\leftarrow H_{b}^{It}\backslash \left\{z\right\}$,34:  **until**
$\mu_{U-S.\;\;A}^{\left(It\right)}\left(b\right)=\mu_{U-S.\;\;A}^{\left(It-1\right)}\left(b\right)$


### 4.2. One-Sided Matching Based Solution Approach Sub-Problem (26)

**Figure 4:**
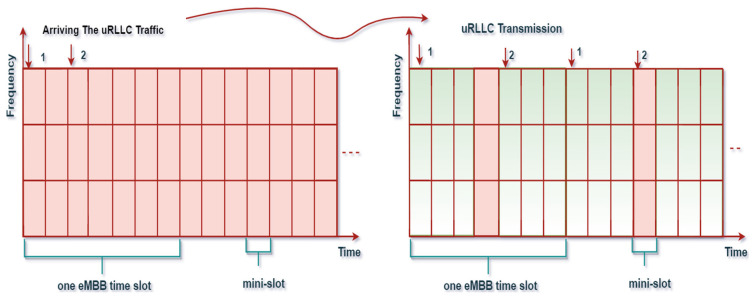
Illustration of the proposed heterogeneous- NS(HNS) pairing.

We can better understand the philosophy of the U−S. P sub-problem and the solution scheme with an interpretative example, as shown in Figure 4. After the association approach, we assume that each BS has sufficient traffic for the connected eMBB and uRLLC UEs. Thus, eMBB users are scheduled on all available PRBs at the beginning of a time slot, and each eMBB UE owns n PRBs long TTI and fixed through the slot. When uRLLC traffic comes abruptly to its associated BS b (in any mini slot of the current slot), the BS b tries to superimpose a uRLLC user with a suitable eMBB user guaranteeing all constraints of each slice and UEs through the mini slot m. Then, the scheduler immediately aims to schedule such traffic with $\rho_{bl}$ paired mini slots. Due to the hard latency bindings of uRLLC traffic, we apply the H-NOMA mechanism to be applied for the uRLLC traffic in this paper. Generally, uRLLC traffic has a small payload size and, thus, requires a fraction of all mini slots for such traffic. However, the questions are about selecting the suitable eMBB and the number of paired mini slots that eMBB users currently occupy are the best to be superimposed, keeping the objective of the sub-problem (26) in mind. Therefore, BS balances the data rate of eMBB users in each long TTI, ultimately achieving the goal of (26) on a long-run basis. In the superimposing scenario of eMBB and uRLLC users associated with the same BS, each uRLLC UE is only able to couple with one eMBB UE per mini slot m, but eMBB UE may be paired with a maximum of $\tilde{\Theta }$ uRLLC UEs per time slot t. Consequently, each uRLLC UE is initially assigned to an eMBB UE. Once this uRLLC UE has been paired with the appropriate eMBB UE, it is deleted, and the procedure is repeated with the remaining set of uRLLC and eMBB UEs. Therefore, we assume the time slot t as two sub-time slots and apply a one-to-one matching game between eMBB and uRLLC users. As for O.P.T1−U−S. P, we erect the preference lists of eMBB and uRLLC UEs based on the following utility functions as $\Lambda_{e}\left(l\right)$ and $\Lambda_{l}\left(e\right)$.

#### 4.2.1. eMBB UEs Utility Function

Firstly, each eMBB UE selects the lowest rate’s uRLLC UE for matching due to owning the entire PRB without a partner. Its preference utility can be represented as:

(34) $$\Lambda_{e}\left(l\right)=R_{bl},$$

#### 4.2.2. uRLLC UEs Utility Function

On the other hand, each uRLLC UE ranks attached eMBB UEs at the same BS with the shortest transmission delay and the highest rate. Thus, each uRLLC UE’s preference utility is dependent on the following:

(35) $$\Lambda_{l}\left(e\right)=R_{be},$$

Algorithm 2 describes the suggested matching-based U−S. P algorithm using the same technique as Algorithm 1. So, as UEs and BS have fixed preference relationships, algorithms are the deferred acceptance algorithm (DAA) for two-sided matching that leads to a stable match [37,42]. We estimate the computational complexity of the described algorithms using the significant O notation. Even under the worst-case scenario, the proposed algorithm’s ongoing complexity may be recognized and compared to other algorithms on both approaches, as shown in Table 2.
**Algorithm 2:** Matching Game for User-Slice Pairing1:  **Input:**
$E_{b},L_{b},\;B$
2:  **Output:** Find stable Matching $\;\mu_{U-S.\;\;P}^{\left(It\right)}$
3:  **Initialization:**
It=0,$\;\mu_{U-S.\;\;P}^{\left(It\right)}={\left\{\mu_{U-S.\;\;P}^{\left(It\right)}\left(l_{b}\right),\;\mu_{U-S.\;\;P\;}^{\left(It\right)}\left(e_{b}\right)\right\}}_{l_{b}\in L,e_{b}\in E,b\in B}=\emptyset ,\;\;\xi_{mp}=\emptyset ,\;z_{l_{b}}^{\left(It\right)}=\emptyset ,\;\tilde{\Theta }=\left\{{\tilde{\Theta }}_{l_{b}}^{rem}=0,{\tilde{\Theta }}_{e_{b}}^{rem}=0,{\tilde{\Theta }}_{e_{b}}^{mod}=0\right\}{\;}_{e_{b}\in E,l_{b}\in L,\;b\in B}$
4:  **Repeat**5:    It←It+1
6:      **for**
 b∈B
**do**7:        Sorts $E_{b}$ attached with b in descending order based on the achieved rate and b construct ${≻}_{l_{b}}$ using $\Lambda_{l_{b}}\left(e_{b}\right)$
8:        **for**
$e_{b}\in E_{\mathrm{sort}}$
**do**9:          $H_{e_{b}}^{It}\leftarrow$ BS b construct ${≻}_{e_{b}}$ using $\Lambda_{e_{b}}\left(l_{b}\right)$
10:          ${\tilde{\Theta }}_{e_{b}}^{rem}\leftarrow$ LbEb11:          ${\tilde{\Theta }}_{e_{b}}^{mod}\leftarrow \left|L_{b}\right|\;mod\;\left|E_{b}\right|$
12:          **If**
${\tilde{\Theta }}_{e_{b}}^{mod}>0$
**do**13:           ${\tilde{\Theta }}_{e_{b}}^{rem}\leftarrow {\tilde{\Theta }}_{e_{b}}^{rem}+1$
14:          **while**
$e_{b}\notin \mu_{U-S.\;\;P}^{\left(It\right)}\left(l_{b}\right)$ and $H_{e_{b}}^{It}\neq \emptyset$
**do**15:            **If**
$\Theta_{l_{b}}^{rem}=$ 0 **then**
16:             $\mu_{U-S.\;\;P}^{\left(It\right)}\left(l_{b}\right)\leftarrow \mu_{U-S.\;\;P}^{\left(It\right)}\left(l_{b}\right)\cup \left\{e_{b}\right\},\;\;$
17:             ${\tilde{\Theta }}_{e_{b}}^{rem}\leftarrow {\tilde{\Theta }}_{e_{b}}^{rem}-1$,18:             $\Theta_{l_{b}}^{rem}\leftarrow \Theta_{l_{b}}^{rem}+1$
19:            **else**
20:             ${\hat{H}}_{l_{b}}^{It}\leftarrow \left\{\hat{e_{b}}\in \mu_{U-S.\;\;P}^{\left(It\right)}\left(e_{b}\right)|e_{b}{≻}_{l_{b}}\hat{e_{b}}\right\},$
21:             $\xi_{mp}$ $\leftarrow \hat{e_{b}}\in {\hat{H}}_{l_{b}}^{It}$22:             **while**
${\hat{H}}_{l_{b}}^{It}\cup$ (${\tilde{\Theta }}_{l_{b}}^{rem}=1$) **do**23:              $\Lambda_{DP}^{\left(It\right)}\left(l_{b}\right)\leftarrow \Lambda_{DP}^{\left(It\right)}\left(l_{b}\right)\backslash \left\{\xi_{mp}\right\}$,24:              ${\hat{H}}_{l_{b}}^{It}\leftarrow {\hat{H}}_{l_{b}}^{It}\backslash \left\{\xi_{mp}\right\}$,25:              $\Theta_{\hat{e_{b}}}^{rem}\leftarrow \Theta_{\hat{e_{b}}}^{rem}+1$
26:              $\Theta_{l_{b}}^{rem}\leftarrow \Theta_{l_{b}}^{rem}-1$
27:              $\xi_{mp}$ $\leftarrow \hat{e_{b}}\in {\hat{H}}_{l_{b}}^{It}$28:              $\Lambda_{DP}^{\left(It\right)}\left(l_{b}\right)\leftarrow \;\Lambda_{DP}^{\left(It\right)}\left(l_{b}\right)\cup \left\{e_{b}\right\},\;\;$
29:  **until**
$\mu_{U-S.\;\;P}^{\left(It\right)}\left(l_{b}\right)=\mu_{U-S.\;\;P}^{\left(It-1\right)}\left(l_{b}\right)$


## 5. Results

### 5.1. Simulation Setup

We consider a two-tier HetNet with four SBSs deployed within a macro cell coverage region radius of 250 m to assess the proposed matching game-based UE-slice association and UE-slice pairing algorithms. SBSs and UEs are uniformly distributed in the coverage area of the MBS. The simulations are dependent on 1000 runs, and the results are averaged. The essential simulation parameters are given in Table 3 [15,54].

### 5.2. Simulation Results

Most research projects on the coexistence between eMBB and uRLLC concur that the ratio of uRLLC to eMBB UEs is double per cell [15,54,58]. So, we assign the quota of U-S. P algorithm as $\tilde{\Theta }=2$. Thus, the paired uRLLC UE superimposes with an eMBB for $\rho_{bl}=3$ mini slots.

The number of eMBB UEs and uRLLC UEs are 25 and 50, respectively [15,54]. The minimum required rate levels of eMBB and uRLLC UEs are 4 and 0.5 Mbps, respectively. The submitted algorithm’s effectiveness is compared with the Early Acceptance algorithm (EAA)-based matching game, Max-SINR, and Greedy algorithm (GA). All these algorithms are displayed in both cases of SINR ${Ґ}_{be}^{nm}$ > ${Ґ}_{bl}^{nm}$ and ${Ґ}_{bl}^{nm}$ > ${Ґ}_{be}^{nm}$, for different techniques (H-NOMA, OMA & Puncturing).

**Figure 5:**
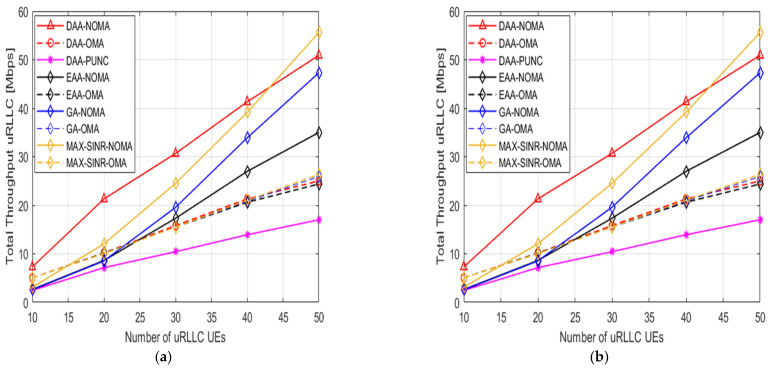
The DL total throughput for uRLLC UEs versus number of uRLLC UEs. (**a**) ${Ґ}_{be}^{nm}$ > ${Ґ}_{bl}^{nm}$. (**b**) ${Ґ}_{bl}^{nm}$ > ${Ґ}_{be}^{nm}$.

We show the total DL throughput of uRLLC UEs with the different numbers of uRLLC when ${Ґ}_{be}^{nm}$ > ${Ґ}_{bl}^{nm}$ and ${Ґ}_{bl}^{nm}$ > ${Ґ}_{be}^{nm}$, in Figure 5 respectively. When uRLLC UEs grow, the uRLLC overall throughput rises in all schemes. In UE-slice association and pairing operations, uRLLC UEs are more interested in latency and reliability than DL data rate. We observe how similar the two shapes are, where our submitted H-NOMA algorithms are still superior in performance over DAA- PUNC and OMA strategies. To explain it, a uRLLC UE is paired with an eMBB UE and occupies the eMBB’s PRBs per $\rho_{bl}$ mini slots. As for using the puncturing technique, an uRLLC UE is overlapped with an eMBB UE and occupies an eMBB’s PRBs per one mini slot. Meanwhile using the OMA technique, each uRLLC UE takes up one PRB per long TTI.

**Figure 6:**
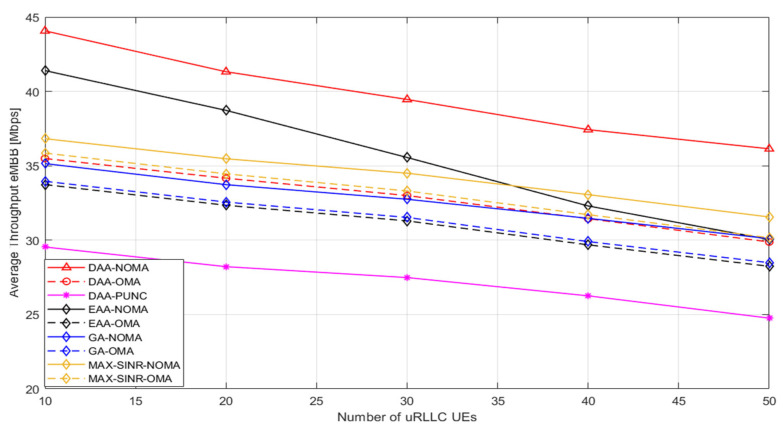
The DL average throughput for eMBB UEs versus number of uRLLC UEs.

After documenting our idea to distribute power among various users’ PRB, it is time to display the other outputs and meet the goals of this study; as shown in Figure 6, the DL average throughput of eMBB UEs is against the number of uRLLC UEs. The average throughput of eMBB UEs falls as the number of uRLLC UEs grows. Even though the DL data rate is not an appealing attachment and pairing metric for uRLLC UEs, it suggests that UEs are vying to associate and pair with the best elected BSs based on their preference lists. The proposed algorithm and EAA-NOMA consider the QoS requirements for eMBB UEs during the association process. Thus, the minimum required DL data rate for eMBB UEs can be achieved, as seen in Figure 6. However, the suggested approach, which incorporates the QoS requirements for eMBB UEs throughout the association and pairing operations, has the lowest rate of loss for eMBB service and outperforms EAA-NOMA, particularly at many uRLLC UEs. This happens because EAA-NOMA does not achieve the best optimum solution since it allows the highest preference list to associate and pair until the system capacity is complete, but the eMBB UEs’ minimum wanted DL data rate is assured; thus, not achieving the spirit of cooperation, which is based on a matching game. Without considering eMBB UE rate needs and the available PRBs at each BS, the performance of both Max-SINR and GA is worse than the proposed algorithm. In Max-SINR, most of the users are associated with MBS.

Meanwhile, SBSs serve a relatively small number of users. Accordingly, many MBS-attached users may not be served because of the limited number of resources available in the MBS. In GA, BSs are unconcerned with the UEs’ QoS requirements and focus only on achieving a higher overall DL rate system. These are explanations for why MAX-SNR and GA have the worst performance rate compared to other schemes.

**Figure 7:**
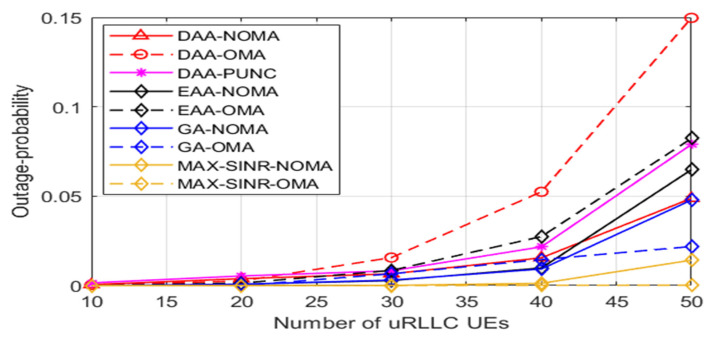
The outage-probability for uRLLC UEs versus number of uRLLC UEs.

Figure 7 illustrates various techniques’ outage probabilities as the number of uRLLC UEs grows. The outage probability is seen in the light as the likelihood that a uRLLC UE consumes latency processes is more than the maximum DL tolerant delay. It can be observed that, at a high density of uRLLC UEs, the outage probability of our submitted scheme stays very low compared to that of PUNC and OMA technology if applied with any algorithm. Simulation results show promising outage probability achievement for uRLLC traffic at various schemes, as shown in Table 4.

**Figure 8:**
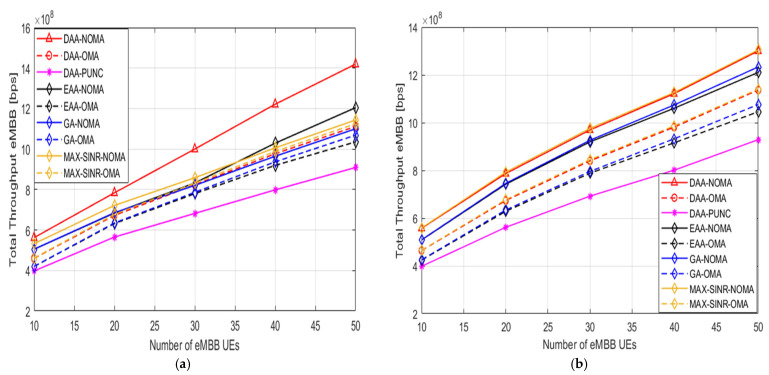
The DL total throughput for eMBB UEs versus number of eMBB UEs. (**a**) ${Ґ}_{be}^{nm}$ > ${Ґ}_{bl}^{nm}$. (**b**) ${Ґ}_{bl}^{nm}$ > ${Ґ}_{be}^{nm}$.

Figure 8 illustrates the overall DL data rate for eMBB UEs when the number of eMBB UEs grows from 10 to 50 and the number of uRLLC UEs = 50 for two cases ${Ґ}_{be}^{nm}$ > ${Ґ}_{bl}^{nm}$ and ${Ґ}_{bl}^{nm}$ > ${Ґ}_{be}^{nm}$, respectively. It can be observed that the total sum rate for eMBB UEs increases if the number of eMBB UEs increases in both cases, but the proposed H-NOMA algorithm demonstrates higher rates if ${Ґ}_{be}^{nm}$ > ${Ґ}_{bl}^{nm}$ (due to its use of higher power and suffering from slight CCI), as shown in Figure 8. Furthermore, the suggested correlation’s performance approach outperforms all other schemes, which affirms the effectiveness of the proposed attachment and pairing approaches because our suggested algorithm considers the type of user, its demands, and intra and inter-isolation constraints. The total sum rate by OMA and Puncturing techniques is lower than that of H-NOMA, indicating that H-NOMA is more sum rate efficient than OMA. The explanations are that, as for the OMA technique, we assume that the transmitted power of an OMA-PRB is $\frac{1}{2}$ the power of a NOMA-PRB; thus, intra and inter-isolation is achieved using the OMA technique. So, each eMBB slice has a fraction of Nb2Nb=45 based on [59]; In the Puncturing technique, when uRLLC UEs are overlapped with eMBB UE, it is scheduled on all eMBB’s PRBs per one mini slot. Simulation results confirm promising gains of up to 40% DL sum rate improvement for eMBB traffic compared to “OMA” and “Puncturing” schemes, as presented in Table 5. So, our assumed framework achieves the highest overall DL data rate for both different users, as illustrated in Figure 5 and Figure 8.

**Figure 9:**
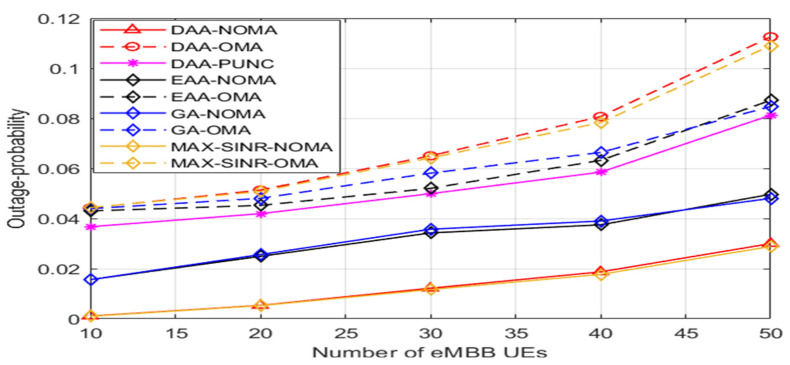
The outage-probability for eMBB UEs versus number of eMBB UEs.

Figure 9 depicts the outage likelihood of the various approaches estimated as the number of eMBB UEs grows. The outage probability of an eMBB UE is represented as not achieving the minimum required DL rate. It can be observed that, at a high density of users, the outage probability of our suggested scheme stays very low compared to other strategies. Simulation results show promising outage probability improvements for eMBB traffic at various schemes, as shown in Table 6.

**Figure 10:**
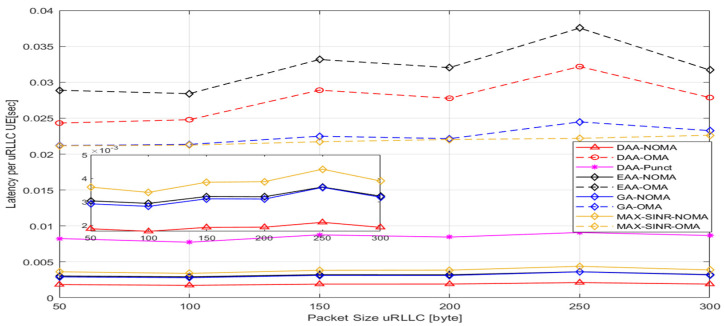
The DL average throughput for eMBB UEs versus offered load uRLLC.

Figure 10 indicates the DL latency per uRLLC as the minimum DL packet size for uRLLC UE increases. This figure proves that the proposed algorithm performs better than the other approaches. Our proposed algorithm proves that each uRLLC packet can be sent in less than 1 msec, regardless of whether it is associated with MBS or SBS, based on the transmission time (backhaul and RAN transmission time), waiting pairing time, and HARQ time. This figure shows that the proposed H-NOMA approach to all algorithms performs better than DAA-PUNC and OMA. The simulation illustrates promising gains of up to 95% and 80% in latency improvement for uRLLC traffic compared to “OMA” and “Puncturing” schemes, respectively, as shown in Table 7.

## 6. Discussion

In the future, further research can be pursued to investigate the following open issues. First, we can use one of the proposed ways to prove its effectiveness in minimizing co-channel interference, such as (a) a hybrid overlay-underlay spectrum access scheme in heterogeneous networks to improve energy efficiency [60]. (b) cooperative relaying networks with NOMA-HetNet [61]. A relay uses a decode-and-forward (DF) scheme to improve the performance of the far user in terms of the outage and throughput. (c) a downlink underlay cognitive radio-NOMA (CR-NOMA) HetNet to reduce CCI and enhance the performance of spectrum sharing [55]. Second, we can combine HetNets with aerially controlled networks such as Unmanned Aerial Vehicles (UAVs) are required to overcome these challenges. UAV-aided HetNet shows lower transmission delay (lower latency) and better average jitter compared to without UAV-based HetNet, because UAVs control the transmission of packets with efficient utilization of available bandwidth from source to destination [62]. Third, we can use FANETs [63], in which multiple UAVs cooperate and establish an ad hoc network in a multi-UAV scenario to enhance network performance. Finally, we will leverage potential deep learning approaches to provide coexisting radio resources for various services. Using a learning-based strategy for flexible joint UE association and pairing will prevent coexistence concerns in 5G and wireless networks.

## 7. Conclusions

In this letter, joint user-slice association and user-slice pairing algorithms for eMBB and uRLLC UEs in HetNet are suggested based on game theory. The suggested algorithms are designed as an optimization problem to maximize the overall DL throughput while assuring the slice’s QoS, minimizing the latency of uRLLC traffic, and achieving intra and inter-isolation using flexible rate isolation. To improve its tractability, we have divided the problem between (1) dynamic association matching of BSs and UEs and (2) superimposing of uRLLC and eMBB UEs. Matching game algorithms for U-S. A and U-S. P are proposed to solve these sub-problems. Moreover, we propose a dynamic eMBB and uRLLC clustering technique called H-NOMA in HetNet systems to balance system performance. The computation complexity for the proposed algorithms is analyzed. Simulation results have confirmed that the submitted algorithm achieved throughput significantly above the compared algorithms and defeated uRLLC UE latency deterioration. The proposed algorithm had the highest throughput and the lowest latency compared with DAA-OMA, DAA-PUNC, EAA-NOMA, GA-NOMA, GA-OMA, MAX-SINR-NOMA, and MAX-SINR-OMA.

## Figures and Tables

**Table 2:** Computational complexity of user-slice association, user-slice pairing schemes.

U-S. A Scheme	Complexity	U-S. P Scheme	Complexity
DAA [37,39,55]	$O\left(UB\right)\;\;$	DAA	$O\left(LE\right)$
EAA [56]	$O\left(UB\log\left(UB\right)\right)$	EAA	$O\left(LE\log\left(LE\right)\right)$
GA [57]	$O\left(U^{2}B^{2}\right)$	GA	$O\left(L^{2}E^{2}\right)$
MAX-SINR	$O\left(U^{2}B^{2}\log\left(UB\right)\right)$	MAX-SINR	$O\left(LE\log\left(E\right)\right)$

**Table 3:** Simulation Parameters.

Parameter	Value
Transmit power of macro-BS	46 dBm
Transmit power of pico-BS	33 dBm
Backhaul bandwidth	40 MHZ
Backhaul timeslot	25 µs
5GRAN bandwidth	20 MHz
Number of PRBs	100
Inter-site distance	500 m
Pathloss between MBS and device	$128.1+37.6*\log\left(d\right)$
Pathloss between SBS and device	$140.7+36.7*\log\left(d\right)$
eMBB-rate threshold	[1−4] Mbps
uRLLC rate threshold	$\left[0.1-1.6\right]$ Mbps
Modulation	4-QAM, 64 QAM for uRLLC and eMBB, respectively
uRLLC packet size	$\left[32-256\right]$ bytes
PHY numerology	15 kHz subcarrier spacing;12 subcarriers per PRB;2-OFDM symbols TTI (0.143 ms)
HARQ	Asynchronous HARQ with chase combining, and 4 TTI round trip time; Max 6 HARQ retransmissions.

**Table 4:** Outage Probability for uRLLC Traffic.

Schemes	Outage Probability
DAA-NOMA	99.04%
DAA-OMA	97.03%
DAA- PUNC	98.3%
EAA-NOMA	98.85%
EAA-OMA	98.64%
GHA-NOMA	99.03%
GHA-OMA	99.42%
MAX-SINR-NOMA	99.78%
MAX-SINR-OMA	99.99%

**Table 5:** DL Sum Rate gains for eMBB Traffic.

Compared Algorithms	DL Sum Rate Gains
DAA-OMA	24.3%
DAA- PUNC	41.9%
EAA-NOMA	16.4%
EAA-OMA	34.3%
GHA-NOMA	18.3%
GHA-OMA	32.3%
MAX-SINR-NOMA	14%
MAX-SINR-OMA	23.6%

**Table 6:** Outage Probability for eMBB Traffic.

Schemes	Outage Probability
DAA-NOMA	98.6%
DAA-OMA	92.9%
DAA-PUNC	94.6%
EAA-NOMA	96.7%
EAA-OMA	94%
GHA-NOMA	96.7%
GHA-OMA	93.9%
MAX-SINR-NOMA	98.6%
MAX-SINR-OMA	93%

**Table 7:** DL latency gains for uRLLC Traffic.

Compared Algorithms	DL Latency Gains
DAA-OMA	92%
DAA- PUNC	77%
EAA-NOMA	40%
EAA-OMA	93%
GHA-NOMA	38%
GHA-OMA	91%
MAX-SINR-NOMA	49%
MAX-SINR-OMA	91%

## Data Availability

The data presented in this study are available on request from the corresponding author.

## Abbreviations

The following abbreviations are used in this manuscript:

RAN	Radio access network
HetNet	heterogeneous networks
NS	Network slicing
QoS	Quality of service
uRLLC	Ultra-reliability low latency communications
eMBB	Enhanced mobile broadband
UE	User equipment
NOMA	Non-orthogonal multiple access
SIC	Successive interference cancellation
U-S. A	UE-slice association
U-S. P	UE-slice Pairing
MBS	Macro base station
SBS	Small bas station
WBH	Wireless backhaul network
H-NOMA	Heterogenous non-orthogonal multiple access
ITU	International Telecommunications Union
mMTC	massive Machine Type Communication
HARQ	Hybrid Automatic Repeat reQuest
CN	Core network
SDN	Software-Defined Networking
NFV	Network Functions Virtualization
EDF	Earliest deadline first
RSMA	Rate-splitting multiple access
CSI	Channel status information
MIMO	multiple-input multiple-output
RIS	Reconfigurable intelligent surface
EPS	Enhanced Pre-emptive Scheduling
C-RAN	Cloud radio access network
DRL	Deep reinforcement learning
MC	Multi-connectivity
PtrNet	Pointer network
RAT	Radio access technology
OFDM	Orthogonal frequency-division multiple access
TTI	Transmission time interval
InP	Infrastructure provider
NLOS	Non-line-of-sight
PRB	Physical resource block
CCI	Co-channel interferences
QoE	Quality of experience
DAA	Deferred acceptance algorithm
EAA	Early acceptance algorithm
MAX-SINR	Maximum-signal-to-interference-plus-noise ratio
GA	Greedy algorithm
OMA	Orthogonal multiple access
PUNC	Puncturing
C-NOMA	Cooperative non-orthogonal multiple access
CR-NOMA	Cognitive radio non-orthogonal multiple access
FANET	Flying Ad Hoc Networks
